# Mitochondrial genome sequences reveal deep divergences among *Anopheles punctulatus* sibling species in Papua New Guinea

**DOI:** 10.1186/1475-2875-12-64

**Published:** 2013-02-14

**Authors:** Kyle Logue, Ernest R Chan, Tenisha Phipps, Scott T Small, Lisa Reimer, Cara Henry-Halldin, Jetsumon Sattabongkot, Peter M Siba, Peter A Zimmerman, David Serre

**Affiliations:** 1 Genomic Medicine Institute, Cleveland Clinic, 9500 Euclid Ave./NE50, 44195, Cleveland, OH, USA; 2 Department of Biology, Case Western Reserve University, 44106, Cleveland, USA; 3 Center for Global Health and Diseases, Case Western Reserve University, Biomedical Research Building, Room E426, 2109 Adelbert Road, 44106-4983, Cleveland, OH, USA; 4 Papua New Guinea Institute of Medical Research, 441, Goroka, Papua New Guinea; 5 Faculty of Tropical Medicine, Mahidol University, 10400, Bangkok, Thailand

**Keywords:** *Anopheles*, *Anopheles punctulatus* sibling species, Molecular evolution, Molecular dating

## Abstract

**Background:**

Members of the *Anopheles punctulatus* group (AP group) are the primary vectors of human malaria in Papua New Guinea. The AP group includes 13 sibling species, most of them morphologically indistinguishable. Understanding why only certain species are able to transmit malaria requires a better comprehension of their evolutionary history. In particular, understanding relationships and divergence times among *Anopheles* species may enable assessing how malaria-related traits (e.g. blood feeding behaviours, vector competence) have evolved.

**Methods:**

DNA sequences of 14 mitochondrial (mt) genomes from five AP sibling species and two species of the *Anopheles dirus* complex of Southeast Asia were sequenced. DNA sequences from all concatenated protein coding genes (10,770 bp) were then analysed using a Bayesian approach to reconstruct phylogenetic relationships and date the divergence of the AP sibling species.

**Results:**

Phylogenetic reconstruction using the concatenated DNA sequence of all mitochondrial protein coding genes indicates that the ancestors of the AP group arrived in Papua New Guinea 25 to 54 million years ago and rapidly diverged to form the current sibling species.

**Conclusion:**

Through evaluation of newly described mt genome sequences, this study has revealed a divergence among members of the AP group in Papua New Guinea that would significantly predate the arrival of humans in this region, 50 thousand years ago. The divergence observed among the mtDNA sequences studied here may have resulted from reproductive isolation during historical changes in sea-level through glacial minima and maxima. This leads to a hypothesis that the AP sibling species have evolved independently for potentially thousands of generations. This suggests that the evolution of many phenotypes, such as insecticide resistance will arise independently in each of the AP sibling species studied here.

## Background

*Anopheles* mosquitoes are distributed worldwide, with the exception of Antarctica, and feed on a variety of hosts from birds to mammals ( [1] and references therein). Within the *Anopheles* genus, 70 of over 500 species are able to transmit human malaria [2]. These include the well-known species *Anopheles gambiae*, *Anopheles arabiensis* and *Anopheles funestus* that are the main vectors of malaria in Africa, *Anopheles darlingi* and *Anopheles albitarsis* in South America, *Anopheles dirus* and *Anopheles minimus* in Southeast Asia (SEA) and *Anopheles punctulatus* in Southwest Pacific (SW Pacific).

Despite their medical importance, the current understanding of the *Anopheles* phylogeny – the relationship among species as well as the times they diverged from each other – remains limited. Studies of this nature are complicated in *Anopheles* by the existence of species complexes (including morphologically identical sibling species) [3-6] and incipient species [7-9], as well as by the paucity of genetic data for most species besides *An*. *gambiae*. The current hypothesis of *Anopheles* evolution, mostly based on the extant geographic distribution of *Anopheles* mosquitoes, proposes that *Anopheles* originated in western Gondwana during the Cretaceous. They subsequently migrated across the world aided by land connections and radiated into multiple species adapted to unique habitats and climatic conditions [10,11]. However, the timing and routes of these dispersions are unknown and the relationships among current species remain poorly understood. Determining the evolutionary relationships among *Anopheles* species has important clinical and vector control implications as it could clarify whether traits required for transmission of human blood-borne pathogens, avoidance of long-lasting insecticide-treated nets or insecticide resistance evolved only once in an ancestral population or, alternatively, whether different species acquired these traits independently.

The focus of this study is on members of the *Anopheles punctulatus* (AP) group, the principal vectors of malaria in Papua New Guinea (PNG), the Solomon Islands, Vanuatu, and northern Australia [12,13]. Historically, the AP group was separated into four species based on morphological differences in the proboscis and wings: *An*. *punctulatus*, *Anopheles koliensis*, *Anopheles farauti*, and *Anopheles clowi*[12]. Later studies involving cross-mating [14-16], allozyme analyses [17] and DNA sequence analysis [18-22] provided evidence suggesting further differentiation of the AP group into 13 species, most of them morphologically indistinguishable. At least five of these species - *An*. *punctulatus* s.s, *An*. *koliensis*, *An*. *farauti* s.s (previously *An*. *farauti* 1), *Anopheles hinesorum* (previously *An*. *farauti 2*), and *An*. *farauti* 4 - have been described as competent vectors of malaria [23,24]. Phylogenetic studies of this group have focused on DNA sequences of ribosomal RNAs [25,26], mitochondrial genes [27,28], ribosomal ITS2 [19], and voltage-gated sodium channel gene [29]. However, the genetic information generated in these studies has not allowed robust determination of the AP group phylogeny and has often yielded conflicting results [19,27]. In addition, no study has yet evaluated the relationships between AP sibling species and other *Anopheles* species from neighbouring regions, such as species from SEA. As Beebe and Cooper have described this group of *Anopheles* sibling species as ‘unspecialized’, there is some reason to hypothesize that AP group members have evolved to acquire a broad range of feeding strategies to ensure survival [30].

Here, the evolutionary history of the AP group of PNG is investigated by sequencing the mitochondrial (mt) genome of 14 individual mosquitoes from the AP group and neighbouring *An*. *dirus* complex. Next generation sequencing technologies were used to generate mt genome sequences and *de novo* assemble each individual sequence. The concatenated sequence of all mitochondrial protein coding genes (10,770 bp) was used to reconstruct robust phylogenies and estimate divergence times among available *Anopheles* mt genomes. The implications of this study’s findings are discussed with regard to the evolutionary history of anophelines in general and the origin of the AP group.

## Methods

### Sampling and laboratory procedures

#### Sample collection and DNA extraction

AP mosquitoes were obtained from the Entomology Unit of the PNG Institute of Medical Research (PNGIMR). *Anopheles dirus* samples were obtained from the Faculty of Tropical Medicine, Mahidol University (Thailand). Genomic DNA from individual mosquitoes was extracted using the Qiagen DNeasy® blood and tissue kit according to the supplementary protocol for purification of insect DNA. The species of each AP mosquito was determined using a PCR-based assay targeting the ITS2 locus [31].

#### Whole genome shotgun sequencing and assembly

The whole genome of five mosquitoes (*An*. *punctulatus* s.s. (n = 1), *An*. *koliensis* (n = 1), *An*. *farauti* s.s. (n = 1), and *An*. *farauti* 4 (n = 2)) were sequenced. Genomic DNA was fragmented into ~300 base pairs and sequencing libraries were prepared using the New England Biolabs (NEB) NEBNext® kit protocol and standard Illumina paired-end adaptors. Each library was sequenced on one lane of an Illumina GAIIx or HiSeq2000 instrument to generate 37 to 150 million paired-end reads from each sample [see Additional file 1].

To identify reads originating from the mt genome and separate them from reads originating from the nuclear genome, the program Bowtie [32] was used to map all reads generated from one sample on the cytochrome oxidase I (COI), cytochrome oxidase II (CO2), and the voltage gated sodium channel (VGSC) gene sequences previously generated for each species. As expected based on the copy number, the sequence coverage of mt genes, COI and CO2, was 50–60 fold greater than the coverage of the nuclear gene, VGSC [see Additional file 2]. The ~500 X coverage of mtDNA implied that multiple identical reads mapped to the exact same nucleotide position along the entire mt genome sequence, therefore, One hypothesis is that most reads occurring twice or more in each shotgun sequencing dataset were likely to originate from the mt genome. These reads were therefore selected (regardless of their DNA sequence) for reconstructing the complete mtDNA sequence of each sample. All assemblies were performed using ABySS [33] with a k-mer size of 29 and C = 70. The assembled contigs were aligned to the mtDNA sequence of *An*. *gambiae* with MUMmer [34] and any remaining gaps were filled by PCR and Sanger sequencing [see Additional file 3]. To identify possible artefacts or assembly errors, all reads generated from a given sample were then mapped to the final mtDNA contig (assembled from a subset of these reads). If necessary, any base in the contig differing from the nucleotide carried by a majority of the reads was replaced by this nucleotide.

#### Multiplexed mt genome sequencing and assembly

For the remaining nine samples, a multiplex approach was used to simultaneously sequence the mt genome [see Additional file 4]. First, primers were designed to amplify any *Anopheles* mt genome using seven overlapping long-range PCRs. An *Anopheles* consensus sequence was generated by aligning the mt genomes of *An*. *punctulatus* s.s., *An*. *farauti* s.s, *An*. *gambiae*[35], *An*. *quadrimaculatus*[36] and *An*. *darlingi*[37] with ClustalW [38] and masking any variant site. Primers were then designed based on this consensus sequence using Primer3 [39] following the Roche Expand Long Range dNTPack® kit recommendations. Primers were able to be designed at overlapping sites with two or less variants and without known variants in the last 3’ positions [see Additional file 3].

Each amplicon was amplified using the Roche Expand Long Range dNTPack® kit protocol with 20–40 ng of gDNA per PCR reaction and 3% DMSO. Amplification conditions were as follows: 3 minute denaturation step at 94°C, 39 cycles of 94°C for 45 seconds, 50°C for 45 seconds, 60°C for 5 minutes followed by a 10 minute final elongation at 60°C. Product amplification was verified by electrophoresis on a 1% agarose gel.

Following amplification, all seven PCR products from a given individual were pooled together and DNA molecules were sheared into 300 bp fragments [see Additional file 4]. A sequencing library for each individual was prepared using Illumina adapters including a unique 6-nucleotide barcode. Finally, libraries were pooled from all mosquitoes in equal concentrations and the resulting pool was sequenced on one lane of an Illumina HiSeq 2000 instrument [Additional file 4], generating an average of 28 million paired-end reads of 100 bp per sample [see Additional file 1] [NCBI SRA: SRP013853].

The mtDNA sequence of each sample was independently assembled. To normalize coverage along the mt genome (necessary to *de novo* assemble the DNA sequence) and remove reads containing sequencing errors, the number of reads carrying each DNA sequence observed was calculated. Given the very high sequence coverage, DNA sequences observed rarely likely correspond to reads with sequencing error(s). Therefore, only DNA sequences represented by >20 reads were used to *de novo* assemble each mt genome. In addition, to normalize coverage and facilitate computation, only one instance of each such sequence was used. This subset of reads was used for *de novo* assembly of the mt genomes using ABySS with a k-mer size of 31. The resulting contigs were aligned to the mtDNA of *An*. *gambiae* using MUMmer. Overlapping contigs were collapsed using nucleotide ambiguity codes in the SeqMan Pro**™** program in DNASTAR’s LASERGENE**®**Core Suite [40] to produce a consensus sequence for each sample. All reads (i.e., not only the subset of reads used to generate the assembly) generated from a given sample were then mapped to its consensus sequence using bwa [41] and the nucleotide determination at each position of the genome sequence was validated using the Samtool mpileup [42] and perl scripts. All mt genome sequences have been deposited in GenBank (see Table 1 for accession numbers). In addition, all complete *Anopheles* mt genomes available from NCBI were retrieved [35-37,43] as well as mt genomes from *Aedes* [GenBank:EU352212.1 and GenBank:AY072044.1], *Culex* [GenBank:HQ724614.1] and *Drosophila*[44,45] (Table 1). Note that six of the 15 mt genomes downloaded from NCBI belong to sibling species of the *An*. *albitarsis* group from South America (*An*. *albitarsis*, *An*. *albitarsis* F, *An*. *albitarsis* G, *Anopheles deaneorum*, *Anopheles janconnae* and *Anopheles oryzalimnetes*).

**Table 1:** List of the samples used in this study with their collection site or colony ID and corresponding NCBI accession numbers

**Species**	**Location**	Length (bp)*	**Reference**	**GenBank No.**
*An. punctulatus* s.s.	Peneng, PNG	15,200	This study	JX219738
*An. punctulatus* s.s.	Dimer, PNG	15,198	This study	JX219737
*An. punctulatus* s.s.	Yagaum, PNG	15,085	This study	JX219739
*An. punctulatus* s.s.	Yagaum, PNG	14,965	This study	JX219740
*An. punctulatus* s.s.	Madang, PNG	15,045	This study	JX219744
*An. farauti* s.s.	Madang, PNG	15,069	This study	JX219741
*An. hinesorum*	Nale, PNG	15,336	This study	JX219734
*An. farauti* 4	Naru, PNG	15,358	This study	JX219735
*An. farauti* 4	Naru, PNG	15,359	This study	JX219736
*An. koliensis*	Nale, PNG	15,113	This study	JX219743
*An. koliensis*	Madang, PNG	15,061	This study	JX219742
*An. dirus* s.s.	Thailand	15,404	This study	JX219731
*An. dirus* s.s.	Thailand	15,126	This study	JX219732
*An. cracens*	Thailand	15,412	This study	JX219733
*An. albitarsis*	Brazil	15,413	[43]	HQ335344.1
*An. albitarsis* F	Columbia	15,418	[43]	HQ335349.1
*An. albitarsis* G	Brazil	15,474	[43]	HQ335346.1
*An. deaneorum*	Brazil	15,424	[43]	HQ335347.1
*An. janconnae*	Brazil	15,425	[43]	HQ335348.1
*An. oryzalimentes*	Brazil	15,422	[43]	HQ335345.1
*An. darlingi* North	Belize	15,386	[37]	GQ918272.1
*An. darlingi* South	Brazil	15,385	[37]	GQ918273.1
*An. quadrimaculatus*	North America	15,455	[36]	L04272.1
*An. gambiae*	G3 strain	15,363	[35]	L20934.1
*Cx. pipiens*	Tunisia	14,856	Unpublished	HQ724614.1
*Ae. aegypti*	unknown	16,655	Unpublished	EU352212.1
*Ae. albopictus*	unknown	16,665	Unpublished	AY072044.1
*D. melanogaster*	United States	19,517	[44]	U37541.1
*D. yakuba*	Ivory Coast	16,019	[45]	X03240.1

^*^ The sequence length reflects the number of actual base pairs assembled (not including Ns).

#### Data analysis

**Phylogenetic analysis and molecular dating** Since relying on the gene annotations from one of the *Anopheles* (i.e. *An*. *gambiae*) may introduce systematic biases in the phylogenetic analyses, the DNA sequence of each gene for each mt genome (i.e. the sequences that were generated as well as those retrieved from NCBI) was determined with tBlastn using the *Drosophila melanogaster* protein annotations as references [Genbank:U37541.1]. Briefly, for each protein coding sequence, the DNA sequences were translated into amino acid sequences, aligned to each other and the amino acid sequence was reverse-translated back into nucleotide sequences with Translator X [46] using the default parameters and the invertebrate mt genetic code. The aligned coding protein sequences from all 13 mt genes (resulting in 10,770 nucleotides) were concatenated and the best model of nucleotide substitutions was determined using the program jModeltest v0.1.1 [47]. According to the Akaike Information Criterion, the best nucleotide substitution model for this data set was the General Time Reversible with gamma distribution (GTR + G) model.

Bayesian phylogenies were reconstructed using BEAST v1.7.2 [48] with the following program parameters: an uncorrelated lognormal relaxed clock model, allowing for rate heterogeneity among species, the GTR + G substitution model, the SRD06 model of partitioning, which allows estimation of nucleotide substitution parameters separately for the 1^st^ + 2^nd^ and 3^rd^ codon positions, and a Yule model for tree reconstruction. Using the above parameters, three independent runs of 20 million generations were performed, with trees sampled every 1,000 generations. All runs were then combined after a burn-in of 10% using LogCombiner v1.7.2. Tracer v1.5 [49] was used to verify adequate mixing of the Markov chains and ensure that each parameter had been appropriately sampled (i.e., effective sampling size >200). The maximum credibility tree was determined using TreeAnnotator v1.7.2 and visualized the phylogenic tree with FigTree v1.3.1 [50].

The program BEAST was used to estimate divergence times using the *Drosophila*-*Anopheles* divergence using a prior distribution normally distributed around a mean of 260 million years ago (mya) and ranging from 243 to 276 mya as suggested in [51]. For comparison, divergence times were also estimated using a mutation rate of 0.0115 mutations per nucleotide per million years, which was estimated from the divergence times and sequence divergence of several insect mt genomes [52].

## Results

### Sequence data and assembly

This study focuses on analysis of the mt genome of 14 individual *Anopheles* mosquitoes from seven species. These include 11 individuals from the AP group in PNG (*An*. *punctulatus* s.s (n = 5), *An*. *koliensis* (n = 2), *An*. *farauti* s.s (n = 1, *An*. *farauti 1*), *An*. *hinesorum* (n = 1, *An*. *farauti* 2), *An*. *farauti 4* (n = 2)) and three samples from the *An*. *dirus* complex in Thailand (*An*. *dirus* s.s (n = 2, *An*. *dirus* species A) and *An*. *cracens* (n = 1, *An*. *dirus* species B) (Table 1).

This study was initiated by sequencing the whole genome of five individual mosquitoes, generating ~37-150 million paired-end reads and resulting in an average 500 X coverage of mtDNA [see Additional file 1 and Additional file 2]. Each individual mt genome was then *de novo* assembled. For nine additional mosquitoes, the mt genome was amplified by overlapping long-range PCR products and all samples were simultaneously sequenced on one lane of an Illumina HiSeq 2000 [see Additional file 4]. This pooled mtDNA sequencing strategy generated 392,983,105 million paired-end reads of 100 bp resulting in an average 188,000 X coverage of each individual mt genome. Each genome was *de novo* assembled separately. For each of the newly sequenced genomes, the genes and gene organization (i.e., orientation and order) were identical to that of previously sequenced *Anopheles* mt genomes [11,35-37,43].

### Phylogenetic analysis

**Figure 1:**
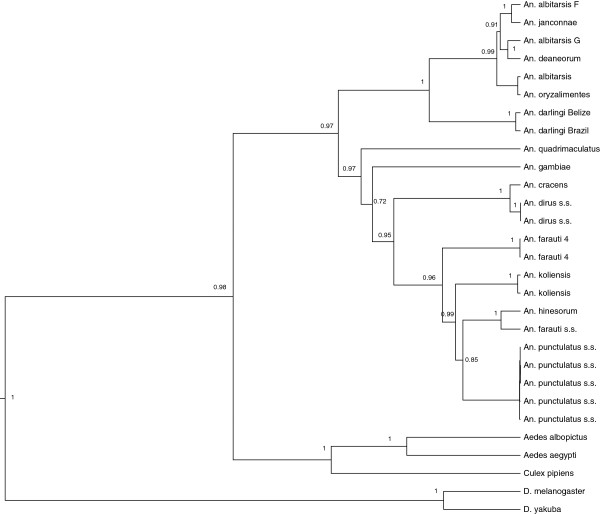
**Support of the *Anopheles *phylogeny using the concatenated DNA sequences of all mitochondrial protein coding genes.** The values on the tree correspond to the posterior probabilities of each node.

The protein coding DNA sequences of all *Anopheles* mt genomes generated here, or previously sequenced, were determined and aligned with several outgroups (Table 1). The concatenated protein coding sequence includes 10, 770 nucleotides. A phylogenetic tree was reconstructed using the concatenated protein coding sequences and the Bayesian approach implemented in BEAST [48]. Three independent runs of 20 million iterations were combined and adequate sampling of the posterior distribution of each parameter was obtained. All phylogenetic relationships were supported with posterior probabilities greater than 90%, with the exception of the position of *An*. *gambiae* (72% support) and an internal node among the AP mosquitoes (85% support) (Figure 1). The resulting phylogenetic tree highlights three monophyletic clades corresponding to the AP, *An*. *dirus* and *An*. *albitarsis* groups (Figure 1).

**Figure 2:**
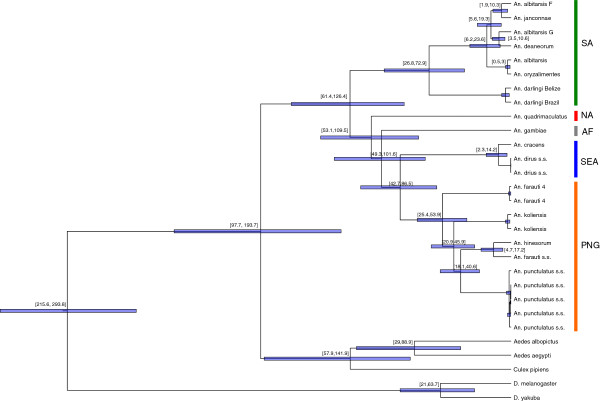
**Phylogenetic tree of *Anopheles *using the concatenated DNA sequences of all mitochondrial protein coding genes.** The bars illustrate the 95% credibility intervals for the divergence times and the numbers in brackets above each node display the actual values in millions of years. The panel on the right indicates the geographic distribution of the samples: the green bar indicates mosquitoes from South America (SA), red from North America (NA), grey from Africa (AF), blue from Southeast Asian (SEA) and orange from Papua New Guinea (PNG).

These results show deep divergence between two main *Anopheles* lineages. One lineage includes all mosquitoes from South and Central America and is further sub-divided into the *An*. *albitarsis* complex and *An*. *darlingi* species. The other lineage, containing all non-Central and South American *Anopheles*, seems to have radiated to generate, first *Anopheles* species currently present in North America and Africa and, from there, SEA and SW Pacific mosquitoes (Figure 2).

The AP group from PNG clusters most closely with the *An*. *dirus* complex distributed across SEA (Figure 2). This tree also partially resolves the phylogeny within the AP group with *An*. *farauti 4* being the most divergent while *An*. *farauti* s.s. and *An*. *hinesorum* being most closely related (Figure 1).

### Molecular dating

As a result of the poor fossil record for mosquitoes [53], few reliable calibration points exist for dating anophelines. The divergence times among *Anopheles* species were therefore estimated using the *Drosophila*-*Anopheles* divergence (260 mya [51]). The most recent common ancestor (MRCA) of all *Anopheles* was dated to 93 mya with a 95% credibility interval ranging from 61 to 126 mya (Table 2). From this origin, *Anopheles* mosquitoes seem to have rapidly diverged from each other and spread across the globe to reach SEA and the SW Pacific by ~43-87 mya (Table 2).

**Table 2:** Mean divergence times and 95% credibility intervals for selected nodes

**MRCA**	**Mean (mya)**	**95% Credibility (mya)**
*Drosophila* / *Anopheles* (Calibration - 260 mya)^a^	255	[215.6-293.8]
Anophelinae / Culicinae	145	[97.7-193.7]
*Anopheles* genus	93	[61.4-126.4]
*An. dirus* complex / *An. punctulatus* group	64	[42.7-86.5]
South and Central American *Anopheles*^b^	47	[26.8-72.9]
*Anopheles punctulatus* group	39	[25.4-53.9]
*Anopheles albitarsis* complex	14	[6.2-23.6]
*An. farauti* s.s./ *An. hinesorum*	10	[4.7-17.2]

^a^ Calibration point. The number in brackets indicates the mean value used for the calibration. The numbers in the table indicates the mean age and 95% credibility interval for this node after analysis.

^b^*An. darlingi* and *An. albitarsis* complex.

Within the AP group, a deep divergence among sibling species with the MRCA of the AP group was observed dating back to 25–54 mya, roughly half as old as the MRCA of all *Anopheles* (Table 2). Importantly, this ancient origin of the AP group does not appear to result from a single highly divergent sibling species: most species analysed here (*An*. *punctulatus* s.s., *An*. *koliensis and An*. *farauti 4*) seem to have diverged 25–54 mya, and only *An*. *farauti* s.s. and *An*. *hinesorum* share a recent common ancestor (5–17 mya) (Figure 2). This finding is especially striking when compared with the only other sequenced *Anopheles* group: *An*. *albitarsis* mosquitoes are distributed across South America (from southern Brazil to Columbia) over a much larger geographic range than the AP group in PNG but share an MRCA dating back to only 6–24 mya, significantly younger than the MRCA of the AP group (Figure 2).

Dating using the estimated insect mt mutation rate of 0.0115 mutations per nucleotide per million years [52] instead of a calibration point, led, overall, to more recent divergence times [see Additional file 5] (but see below).

## Discussion

The predominant hypothesis regarding the origin of *Anopheles* mosquitoes predicts that they originated in western Gondwana [10,11] and that, by 95 mya, *Anopheles* had migrated into Africa. Ancestral *Anopheles* are predicted to have then colonized Europe and North America (via land bridges), and migrated through Asia into the SW Pacific. The topology of the tree in Figure 2 is globally consistent with this hypothesis. However, the position of North American mosquitoes (*An*. *quadrimaculatus*) relative to African and other non-American *Anopheles* remains unclear. In particular the lack of mt genomes from European *Anopheles* mt genomes preclude determining whether African *Anopheles* are ancestral to European and North-American *Anopheles* or, alternatively, whether North-American *Anopheles* derive directly from South-American *Anopheles*. Additional sampling of mt genomes would provide better resolution of *Anopheles* early dispersal routes.

Regarding SEA and the SW Pacific, it is generally believed that *Anopheles* from the SW Pacific derives from SEA mosquitoes [10,26,27]. The results of this study suggest that the AP group is most closely related to the *An*. *dirus* complex of SEA, consistent with an origin of the AP group in SEA. However, currently it cannot be determined whether SW Pacific and Australian *Anopheles* originate from an SEA ancestor as currently hypothesized or, alternatively, whether SW Pacific and SEA *Anopheles* have an Australian origin. The molecular dates in this study, suggest that the ancestor of the AP group was present in PNG between 25 and 54 mya but does not allow rejecting either of these scenarios. Plate tectonic models show that the Australia/PNG plate, that separated from Gondwana during the Cretaceous, moved from a southern position in the Eocene to its current position near SEA [54]. While the upper limit of the age of the AP ancestor (54 mya) corresponds to a time when the distance between PNG and SEA would not have allowed migration between these regions, the lower limit (~25 mya) corresponds to a time when the Australian plate had moved close enough to the Asian plate to enable possible migration of species between the two regions [55]. Inclusion of additional mt genome sequences, in particular from *Anopheles* complexes restricted to Australia (e.g., *Anopheles annulipes*) may allow better understanding of these early dispersal routes in SEA and SW Pacific.

The monophyly of AP mosquitoes (Figure 1) suggests that they colonized PNG through a single migration event followed by speciation (as opposed to multiple migrations of pre-established species). This study suggests that the different AP sibling species diverged from each other 25–54 mya, much earlier than proposed in previous studies of the AP group [26,28]. This deep divergence among AP mosquitoes is unlikely to be caused by a single species that could have diverged from the other sibling species in SEA and colonized PNG later. In fact, most of the AP sibling species are equally divergent from each other, suggesting rapid radiations of AP sibling species upon arrival in the SW Pacific area. Since this analysis relies on a single non-recombining locus, it cannot rule out the possibility that the estimates are influenced by the action of natural selection. However, when only *Anopheles* mt genomes are analysed, there is little evidence for deviation from a clock-like model of evolution, which suggests that nucleotide substitutions occur at a similar rate on each lineage. Therefore, if natural selection is driving the evolution of the mt genome in *Anopheles*, it is likely to have acted in a similar manner on all lineages and consequently is unlikely to bias the molecular dates significantly. An additional possible complication is that the phylogenetic tree inferred from mt sequences differs from the actual species tree: since these analyses are based on a single locus, one cannot rule out that incomplete lineage sorting and introgression lead to a phylogenetic reconstruction that does not represent the true evolutionary pathways of the species studied [56,57]. The long internal branches separating species coupled with the very short branches separating individuals from the same species indicate that incomplete sorting of ancestral polymorphisms is unlikely to affect this phylogeny [58]. Ruling out introgression is difficult without genetic data on multiple unlinked loci (i.e., nuclear), which are complicated to generate due to the lack of a good reference genome sequence (*An*. *gambiae* being too divergent), the high divergence among AP species at the nuclear level and the high level of genetic diversity within species. However, there is little evidence of gene flow among AP mosquitoes (but see also Ambrose [28]) and early forced mating studies suggested that most hybrids are non-viable or sterile [15]. Overall, these observations suggest that, for this dataset, the mt gene tree likely recapitulates the actual species tree.

**Figure 3:**
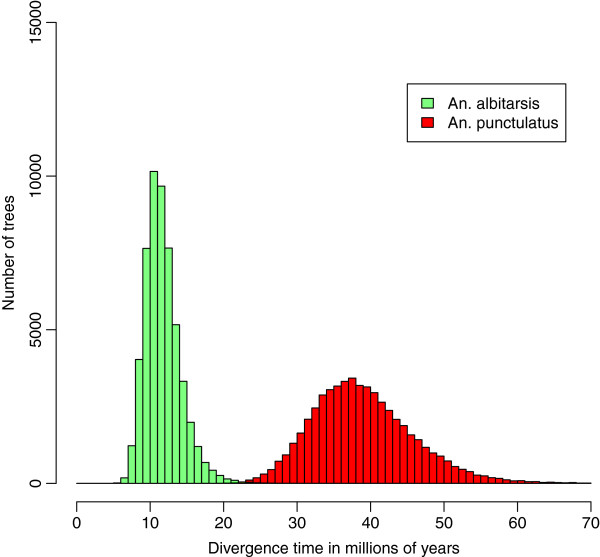
**Distribution of all sampled divergence times for the MRCA of the *An*. *albitarsis *(green) and *An*. *punctulatus *(red) groups obtained using BEAST (after burn-in)**

Absolute dates estimated from molecular data should be consider cautiously, especially since these estimates rely on a single calibration point. Note however that the divergence estimates between *An*. *quadrimaculatus* and non-American *Anopheles*, as well as, between *An*. *farauti* s.s. and *An*. *hinesorum*, are similar to those of previous studies [11,28,37]. When the estimated mutation rate for insect mtDNA was used [52], the divergence dates obtained were significantly younger than those obtained using a calibration point [see Additional file 5]. This mutation rate, while widely used (see e.g. [28]) was originally calculated using closely related species (the maximum divergence time used in the study was ~3 mya) and, Brower noted, probably overestimated the actual mutation rate. A slower mutation rate would push estimated divergence dates back in time, closer to the estimates obtained using calibration points. Note that even considering these younger divergence dates, the MRCA of AP mosquitoes would still considerably predate the arrival of humans in PNG (see below). Irrespective of the absolute dates, it is interesting to note that the AP group is significantly older than the only other *Anopheles* group for which several mt genome sequences are available, the *An*. *albitarsis* complex (Figure 3). Indeed, the MRCA of the AP group is estimated to be approximately four times older (25–54 mya) than the *An*. *albitarsis* complex MRCA (6–24 mya) and almost half as old as the MRCA of all *Anopheles* mosquitoes. Based on these findings, it can be speculated that after arriving in PNG 25 to 54 mya, the ancestors of AP group became isolated on different islands as sea levels fluctuated and their distribution only overlapped later, when the sea level decreased to merge different islands into current-day PNG.

Given the old divergence times among most AP sibling species, one would expect that most AP species today are reproductively isolated and that hybridization is unlikely to occur in nature, with the possible exception of *An*. *farauti* s.s and *An*. *hinesorum* that only diverged 5 to 17 mya (see also [28]). This potential reproductive isolation among AP sibling species is supported by early cross-mating experiments suggesting that F1 hybrids between any combination of *An*. *farauti* s.s, *An*. *hinesorum*, *An*. *koliensis* and *An*. *punctulatus* s.s are non-viable or sterile in laboratory [15]. These results have important implications for vector control in the SW Pacific. For example, control of malaria by releasing genetically modified-, sterile-, male mosquitoes as was recently proposed [59] would require, in PNG, independent engineering of mosquitoes from, at least, five highly divergent species to significantly impact the populations of the main malaria vectors. In addition, if AP sibling species are reproductively isolated from each other, insecticide resistance arising in one species is unlikely to spread quickly across all AP mosquitoes, but instead resistance mutations would have to occur independently in each species [29]. Further investigations are required to definitively rule out the existence of gene flow among most AP sibling species, as well as to confirm the observation of putative mt genome introgression between *An*. *farauti* s.s and *An*. *hinesorum* recently described in Southern New Guinea [28].

The ancient divergences among *Anopheles* species, including among AP from SW Pacific, also raises questions for the evolution of traits related to human malaria transmission. Transmission of *plasmodium* parasites to humans is facilitated by the anthropophilic behaviour of some *Anopheles* species. Such behaviour includes usage of human-made habitats as breeding sites and preferential feeding on human blood. Some *Anopheles* mosquitoes, including most populations of *An*. *gambiae*, are highly specialized feeders and rely preferentially on humans for their blood meal [60]. Others are considered more “opportunistic” or “generalist” and the source of their blood meal varies according to several factors including host density (for review see [61]). There are limited data on the feeding preference and behaviour of AP mosquitoes. Early studies of AP mosquitoes seem to indicate a relatively unspecialized feeding behaviour [62,63].

On the other hand, recent studies by Cooper and colleagues have shown that at least two populations of *An*. *hinesorum* show strong feeding preferences and specifically avoid biting humans [22,28]. Frequency of utilization of human-made habitats for breeding also varies among the AP sibling species [22]. The phylogenetic study described here suggests that the AP sibling species diverged from each other long before humans arrived in PNG, probably ~50,000 years ago [64]. In fact, the molecular dates place the divergence time of AP mosquitoes before the emergence of the Hominidae (or great ape) family [65,66]. This indicates that any adaptations to humans (for blood meal or larval habitats) would have occurred independently in each AP sibling species, rather than being inherited from a common ancestor, and suggests that co-occurrences of malaria-related traits in *Anopheles* are the results of convergent evolution [67,68].

## Conclusions

The ancient divergence among AP sibling species, coupled with the recent arrival of humans in PNG, indicates that AP mosquitoes were present in PNG long before humans colonized the island. This observation suggests that the AP mosquitoes have independently evolved to adapt their behaviour to humans. While further studies are needed to better characterize the behaviour of AP mosquitoes, these findings emphasize the potential of the AP group to serve as a model for studying the evolution of vector competence and potentially for identifying the genetic basis of the ability to transmit human malaria.

## Competing interests

The authors declare that they have no competing interests.

## Authors’ contribution

PAZ and DS designed the research, KL, EC and TP performed the experiments and analysed the data, LR, CHH, STS, JS, PMS, PAZ and DS contributed new reagents or analytical tools, KL, EC, STS, PZ and DS wrote the paper with insights from all other co-authors. All authors read and approve the final manuscript.

## Supplementary Material

Additional file 1 **Sample sequencing information.** List of samples sequenced in this study, their sequencing method, read length and the number of paired-end reads generated. Click here for file

Additional file 2 **Coverage of whole genome sequencing reads on mitochondrial and nuclear genes.** Coverage per base pair of whole genome sequencing read pairs mapped to 2 mitochondrial genes (COX2 and COI) and one nuclear gene (VGSC). Each color represents one of the mapped read pairs. The numbers in the center of each bar represent the actual coverage per base pair. Click here for file

Additional file 3 **Primers used in study.** Primers used to amplify mitochondrial genomes by long range PCR, to fill-in gaps between assembled contigs from whole genome sequencing of *An*. *punctulatus* and *An*. *farauti* 1 (51 bp reads), and to amplify the control region (A + T rich region) of several mitochondrial genomes. Nucleotides in red indicate variable sites among *Anopheles*. Click here for file

Additional file 4 **Multiplex sequencing method.** Diagram of the steps used to amplify and sequence multiple mitochondrial genomes simultaneously on one lane of an Illumina Hiseq 2000 instrument after amplification by long range PCR. Click here for file

Additional file 5 **Divergence times using the insect mitochondrial DNA mutation rate.** Mean divergence times and 95% credibility intervals for selected nodes using insect mitochondrial DNA mutation rate. Click here for file
