# Supplementary material for: Mitochondrial genome sequences reveal deep divergences among Anopheles punctulatus sibling species in Papua New Guinea
Source: Malar J. 2013 Feb 14;12:64. doi: 10.1186/1475-2875-12-64 (PMC3577438; doi:10.1186/1475-2875-12-64)
Supplement: Additional file 1 — Sample sequencing information. List of samples sequenced in this study, their sequencing method, read length and the number of paired-end reads generated. [file 1475-2875-12-64-S1.doc]

| Sample | Seq Method | Sequencer | Read length | Number of Read Pairs |
| --- | --- | --- | --- | --- |
| *An. punctulatus* s.s. | Whole Genome^a^ | Illumina GAIIx | 51 | 39,399,308 |
| *An. farauti* s.s. | Whole Genome | Illumina GAIIx | 51 | 44,281,276 |
| *An. farauti* 4 | Whole Genome | Illumina HiSeq 2000 | 100 | 122,694,078 |
| *An. farauti* 4 | Whole Genome | Illumina HiSeq 2000 | 100 | 150,366,415 |
| *An. koliensis* | Whole Genome | Illumina GAIIx | 58 | 37,073,523 |
| *An. punctulatus* s.s. | Multiplex^b^ | Illumina HiSeq 2000 | 100 | 11,764,434 |
| *An. punctulatus* s.s. | Multiplex | Illumina HiSeq 2000 | 100 | 17,419,685 |
| *An. punctulatus* s.s. | Multiplex | Illumina HiSeq 2000 | 100 | 13,082,863 |
| *An. punctulatus* s.s. | Multiplex | Illumina HiSeq 2000 | 100 | 10,495,346 |
| *An. hinesorum* | Multiplex | Illumina HiSeq 2000 | 100 | 20,253,194 |
| *An. koliensis* | Multiplex | Illumina HiSeq 2000 | 100 | 15,527,845 |
| *An. dirus* s.s | Multiplex | Illumina HiSeq 2000 | 100 | 13,399,633 |
| *An. dirus* s.s | Multiplex | Illumina HiSeq 2000 | 100 | 13,431,487 |
| *An. cracens* | Multiplex | Illumina HiSeq 2000 | 100 | 11,527,398 |

^a^Whole genome sequence of one individual mosquito.

^b^All Multiplex sequences were given a unique barcode and pooled together for sequencing on one lane of a flow cell.
