# Supplementary figures and images for: Mitochondrial genome sequences reveal deep divergences among Anopheles punctulatus sibling species in Papua New Guinea
Source: Malar J. 2013 Feb 14;12:64. doi: 10.1186/1475-2875-12-64 (PMC3577438; doi:10.1186/1475-2875-12-64)

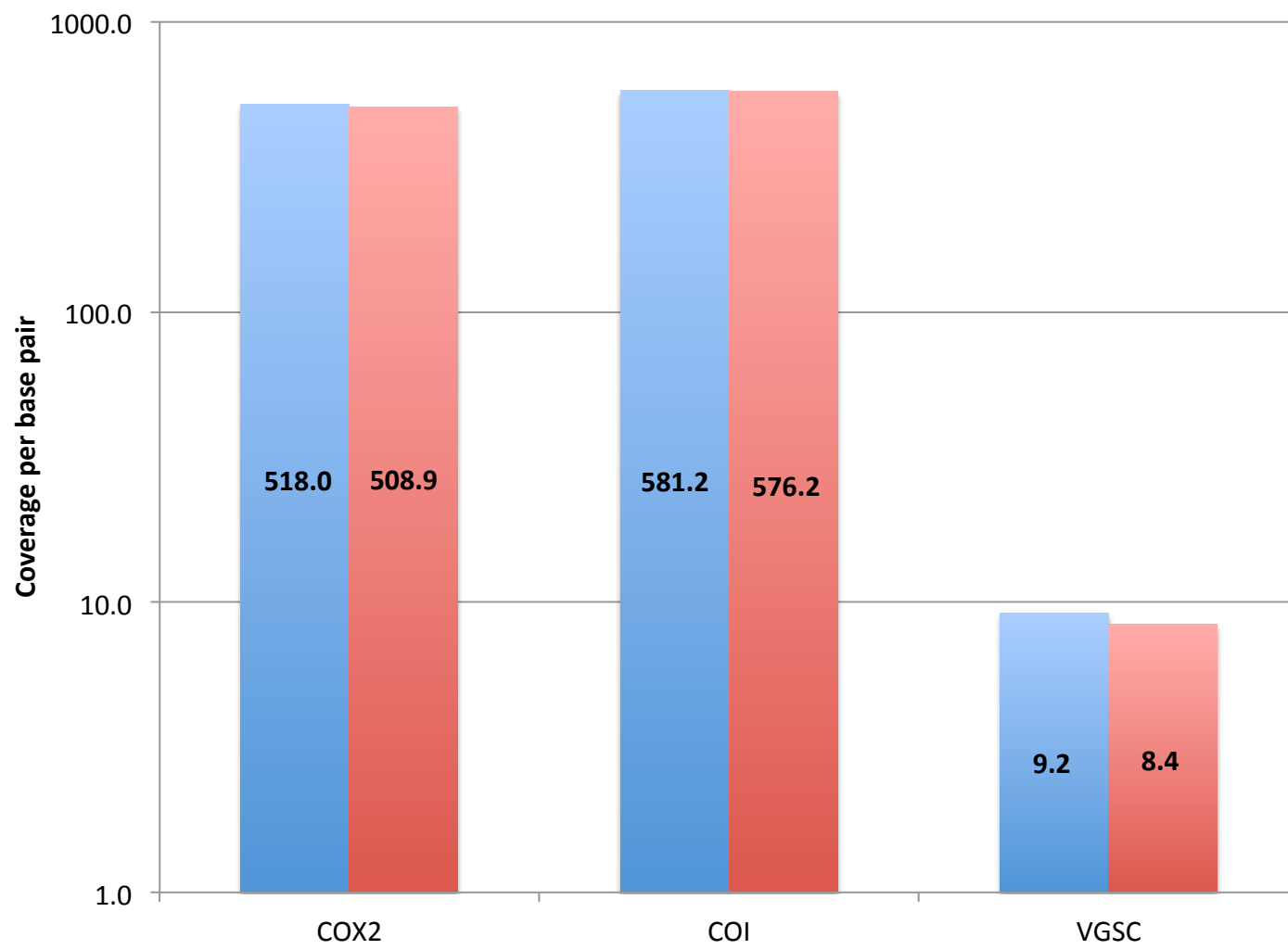

Supplement: Additional file 2 — Coverage of whole genome sequencing reads on mitochondrial and nuclear genes. Coverage per base pair of whole genome sequencing read pairs mapped to 2 mitochondrial genes (COX2 and COI) and one nuclear gene (VGSC). Each color represents one of the mapped read pairs. The numbers in the center of each bar represent the actual coverage per base pair. [file 1475-2875-12-64-S2.pdf]

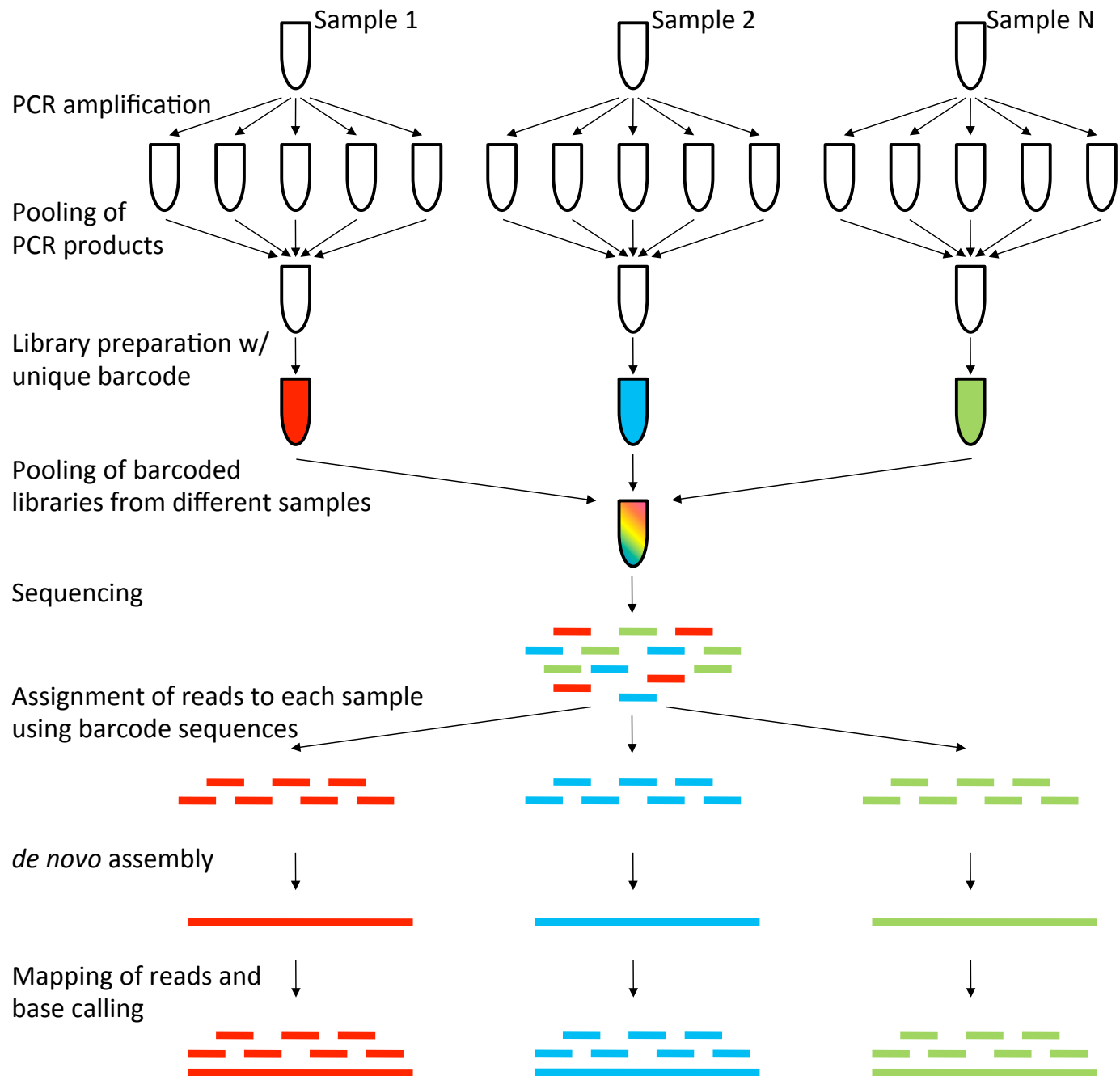

Supplement: Additional file 4 — Multiplex sequencing method. Diagram of the steps used to amplify and sequence multiple mitochondrial genomes simultaneously on one lane of an Illumina Hiseq 2000 instrument after amplification by long range PCR. [file 1475-2875-12-64-S4.pdf]
