# Supplementary material for: Mitochondrial genome sequences reveal deep divergences among Anopheles punctulatus sibling species in Papua New Guinea
Source: Malar J. 2013 Feb 14;12:64. doi: 10.1186/1475-2875-12-64 (PMC3577438; doi:10.1186/1475-2875-12-64)
Supplement: Additional file 3 — Primers used in study. Primers used to amplify mitochondrial genomes by long range PCR, to fill-in gaps between assembled contigs from whole genome sequencing of An. punctulatus and An. farauti 1 (51 bp reads), and to amplify the control region (A + T rich region) of several mitochondrial genomes. Nucleotides in red indicate variable sites among Anopheles. [file 1475-2875-12-64-S3.doc]

| **Primer Name** | **Sequence** | **Length (bp)** |
| --- | --- | --- |
| **Long Range PCR of Mitochondrial genomes** | | |
| 133_3048F | AAAAAGATAAGCTAA**TT**AAGCTATTGG | 27 |
| 133_3048R | TAAAGGAGAAGAACTATCTTGTAA**T**CC | 27 |
| 3032_5516F | AACATGAGCAAATTTAGG**A**TTACAAG | 26 |
| 3032_5516R | AAATT**A**TTTAGTCCTTGTGATTGGAAG | 27 |
| 5507_7060F | TATATGTGACTTCCAATCACAAGGAC | 26 |
| 5507_7060R | ATATTGATTTGTGGTGTCAATGATATG | 27 |
| 5952_10891F | GAAATTCACCCATATTTTAGGGTAATAG | 28 |
| 5952_10891R | CAAATCC**T**CCTCAAATTCATTG | 22 |
| 10771_13066F | TTAAC**A**ATAGC**A**ACAGGATTTTTAGG | 26 |
| 10771_13066R | AAAAT**A**TAATTAAA**G**GACGAGAAGACC | 27 |
| 12814_14383F | TAAAAATAA**C**TCTTAATCCAACATCGAG | 28 |
| 12814_14383R | CGGTGTTTTAGTCTATTTAGAGGAATC | 27 |
| 14234_745F | TACTTAA**AT**ATAAACTGCACCTTGACC | 27 |
| 14234_745R | TTATTGCTAATA**AA**ATTCATCCTAAATG | 28 |
| **Amplification of gaps from Illumina GAIIx whole genome sequencing** | | |
| mtAF7aF | CGCAGTAGCTGGCACAAAT | 19 |
| mtAF7aR | TCCTTTTTATCAGGCAATTCA | 21 |
| mtAP2aF | TGCCGAATTCTTCATTAAAACC | 22 |
| MtAP2aR | AAAACACCGCCAAATTCTTT | 20 |
| MtAP3aF | TTGTACCTTGTGTATCAGGGTTT | 23 |
| MtAF5aF | GAATTAGAAGACCATCCAGCAA | 22 |
| MtAF6aF | GCGGCCCTTTAAATTTCAGT | 20 |
| **Amplification of Control Region** | | |
| APsibsCtrlF | CGCAGTAGCTGGCACAAAT | 19 |
| APCtrlR | AACCCTTTTATCAGGCAATTCA | 22 |
| APsibsCtrlR | TCCTTTTTATCAGGCAATTCA | 21 |
| AF1CtrlF | CCATTTGTATAACCGCAGTAGC | 22 |
| AF1CtrlR | TTTCATGATTTACCCTATCAAGG | 23 |
| ADBCtrlR | TCCTTTTTATCAGGCAATCCA | 21 |
