# Supplementary material for: Mitochondrial genome sequences reveal deep divergences among Anopheles punctulatus sibling species in Papua New Guinea
Source: Malar J. 2013 Feb 14;12:64. doi: 10.1186/1475-2875-12-64 (PMC3577438; doi:10.1186/1475-2875-12-64)
Supplement: Additional file 5 — Divergence times using the insect mitochondrial DNA mutation rate. Mean divergence times and 95% credibility intervals for selected nodes using insect mitochondrial DNA mutation rate. [file 1475-2875-12-64-S5.doc]

| MRCA | Mean (mya) | 95% Credibility (mya) |
| --- | --- | --- |
| *Drosophila* / *Anopheles* | 80.5 | [59.7-104.1] |
| Anophelinae / Culicinae | 46.1 | [37.9-55.4] |
| *Drosophila* | 12.7 | [8.2-18.2] |
| *Anopheles* genus | 29.3 | [24.6-34.9] |
| *An. dirus* complex / *An. punctulatus* group | 20.4 | [16.4-24.6] |
| South and Central American *Anopheles*^b^ | 14.5 | [10.3-19.1] |
| *An. punctulatus* group | 12.5 | [10.2-15.4] |
| *An. albitarsis* complex | 3.8 | [3.0-4.8] |
| *An. farauti* s.s. / *An.hinesorum* | 3.1 | [2.0-4.5] |
